# A Case Report: Lithium-Induced Neurotoxicity, a Differential to Always Consider

**DOI:** 10.7759/cureus.50225

**Published:** 2023-12-09

**Authors:** Noah N Kronk, Brooke K Kronk, Ahmed T Robbie

**Affiliations:** 1 Emergency Medicine, University of Missouri School of Medicine, Columbia, USA; 2 Neurology, University of Missouri School of Medicine, Columbia, USA; 3 Neurology, Mercy Hospital, Springfield, USA

**Keywords:** chronic kidney disease (ckd), family medicine, adult neurology, emergency medicine, lithium toxicity, chronic lithium therapy

## Abstract

Lithium, a mood stabilizer commonly prescribed for bipolar disorder, has a narrow therapeutic index that increases the risk of toxicity for patients who are prescribed this medication. Patients presenting with lithium toxicity could have a wide array of symptoms triggered by several factors that mimic other neurological conditions. In this paper, we discuss the case of an 81-year-old male who presented to the emergency department with worsening tremors and visual hallucinations, ataxia, and cognitive decline. He was initially thought to have Parkinson's disease with dementia in the outpatient setting and was later found to have lithium toxicity. Swift identification and management, involving fluid diuresis, led to the complete resolution of the patient's neurological symptoms by the fourth day of hospitalization. This case calls attention to the challenges of diagnosing lithium toxicity due to the variability in presentation as well as precipitating factors that clinicians must be cognizant of when working up patients who are prescribed lithium.

## Introduction

Lithium toxicity was first reported in the literature in 1949 when lithium chloride was used in patients with heart failure as a substitution for salt [1]. Approximately 6,000 to 7,000 instances of lithium intoxication are annually reported to the American Association of Poison Control Centers [2]. Due to its narrow therapeutic range, a significant portion of patients undergoing chronic lithium therapy typically encounter at least one instance of toxicity throughout their treatment [3].

Lithium toxicity may arise from intentional or accidental overdose, as well as from recent medication adjustments or underlying conditions that result in reduced renal function due to hypovolemia. Elderly individuals tend to experience more pronounced chronic toxicity from lithium, even when prescribed lower doses compared to younger adults. This is often attributed to age-related reductions in glomerular filtration rate and diminished volume of distribution, which can be linked to declines in lean body mass and total body water [4].

The side effects of lithium vary based on the duration of exposure. Acute toxicity is primarily linked to gastrointestinal symptoms, such as diarrhea, nausea, and vomiting, whereas chronic toxicity is more characterized by neurological manifestations, including tremor, agitation, confusion, neuromuscular excitability, and ataxia.

This case report details a patient on chronic lithium therapy who underwent a recent change in his medication regimen before experiencing a deterioration in symptoms. The patient was initially suspected to have a distinct neurological diagnosis during prior outpatient evaluations but was ultimately determined to have lithium toxicity.

## Case presentation

An 81-year-old male with a past medical history of bipolar depression on lithium for 20 years, well-controlled hypertension, congestive heart failure, and stable stage 3 chronic kidney disease presented to the emergency department with progressively worsening resting tremor, ataxia, confusion, and cognitive decline for six weeks. According to his family, approximately six weeks ago, he underwent an evaluation at the cardiology clinic, where it was determined that his heart failure had worsened, resulting in an ejection fraction of 35-40%. This was a decrease from the ejection fraction of 55% that was measured one year earlier. Subsequently, he was initiated on sacubitril-valsartan 24-26 mg (Entresto), followed by the addition of spironolactone 25 mg one week later. During the ensuing three weeks, he began to notice a worsening of resting tremors and visual hallucinations, both of which had originally manifested the previous year. In addition, his family reported he was experiencing confusion and disorientation, raising concerns about possible dementia. He was seen by his primary care physician where a comprehensive metabolic panel, complete blood count, and urinalysis were obtained, all of which returned unremarkable. That same day he started on carbidopa/levodopa 25-100 mg tablet 3x daily due to concerns of Parkinson's disease dementia. In the following three weeks, his family reported that the tremors continued to worsen and he began having auditory and visual hallucinations. His gait became worse, and he was unable to use his walker to help get himself around the house. Ultimately, he refrained from both eating and drinking, prompting his family to choose to take him to the emergency department for a thorough evaluation.

Upon arrival at the emergency department, the patient's height and weight were recorded at 177.8 cm and 103 kg, respectively. A physical exam in the emergency department showed a resting tremor in the left hand but no other abnormalities were appreciated. A neurological exam showed altered mental status, and the patient was unable to answer most questions appropriately. The patient was unable to form comprehensible sentences and was unable to tell the medical staff his name or current location. His resting tremor was found to be worse with finger-to-nose testing, and he had rigidity in the arms and legs. Initial laboratory workup in the emergency department (Table 1) consisted of a complete blood count (CBC) and comprehensive metabolic panel (CMP), magnesium, phosphorus, and thyroid-stimulating hormone (TSH). Additional testing included an EKG 12-lead, a non-contrast CT head, and a neurology consult. EKG interpretation showed an abnormal ECG with nonspecific ST-T wave changes. The neurology team recommended adding on a lithium level and brain MRI. Head imaging showed no abnormalities and lithium level was found to be 1.8 mmol/L. Lithium was withheld from his medications and the dosage was reviewed and noted to be 300 mg BID. The patient was then admitted to the general internal medicine floor and nephrology was consulted. The recommendation was given to initiate diuresis via IV fluids and dialysis was not indicated at that time.

**Table 1:** Summary of initial laboratory workup in the emergency department

Parameter	Value	Reference Range
Hemoglobin (g/dL)	13.8	14.0-18.0
White blood cell count (K/uL)	9.5	4.8-10.8
Sodium (mmol/L)	142	135-146
Potassium (mmol/L)	4.9	3.5-5.3
Chloride (mmol/L)	110	98-110
CO2 (mmol/L)	23	20.0-32.0
Calcium (mg/dL)	10.6	8.6-10.3
Albumin (g/dL)	4.2	3.6-5.1
Blood urea nitrogen (BUN) (mg/dL)	35	7.0-25.0
Creatinine (mg/dL)	2.11	0.70-1.22
Blood glucose level (mg/dL)	106	65.0-99.0
Alanine aminotransferase (U/L)	<5	<=50
Aspartate aminotransferase (U/L)	13	10-50
Magnesium (mg/dL)	2.5	1.6-2.4
Phosphorous (mg/dL)	3.0	2.5-4.5
Thyroid-stimulating hormone (TSH) (uIU/ml)	2.88	0.27-4.20
Serum lithium level (mmol/L)	1.8	0.6-1.2

Over the following two days, the patient was diuresed via IV fluids, and his lithium level normalized by day three of admission. The morning laboratory tests revealed an elevated sodium level of 149 mmol/L, which had risen from 142 mmol/L the previous day. This level continued to climb, reaching 153 mmol/L on the following day. Partial lithium-induced nephrogenic diabetes insipidus was suspected and nephrology recommended starting dextrose 5% in water. By the fourth day of his hospital admission, the patient's tremors had completely disappeared, and his mental state had returned to its baseline. He was able to respond to questions and engage in conversations appropriately. His auditory hallucinations were no longer present and his visual hallucinations were becoming less frequent. By the eighth day of the hospital course, the patient's sodium level returned to normal, leading to the discontinuation of dextrose 5% in water. The patient was encouraged to increase free water intake and their sodium level remained within normal limits during the rest of their hospital stay. On the tenth day of hospitalization, the patient's kidney function had returned to its baseline state. Lithium was removed from his medication regimen, and on the eleventh day of his hospital stay, he was discharged to return home. He was prescribed lamotrigine at a dosage of 50 mg twice daily, with gradual weekly increases, and was scheduled for outpatient management with a psychiatry follow-up appointment later in the same month.

## Discussion

In the presented case, the initial diagnosis of lithium toxicity in the emergency department was made by evaluating the patient's clinical history, physical examination findings, and a serum lithium level of 1.8 mmol/L. Before this diagnosis was confirmed, there was uncertainty regarding whether the patient's signs and symptoms were related to lithium toxicity or the progression of a previously assumed diagnosis of Parkinson's disease dementia that had been tentatively established during outpatient visits. Upon reviewing the patient's medical records, it was discovered that the patient had never previously exhibited a lithium level exceeding 0.9 mmol/L, further supporting the argument that the patient's presentation was indeed a result of lithium toxicity. Because lithium has a narrow therapeutic range, it is advised to undergo monitoring both in the acute phase and the stabilization phase. In the stabilization phase, it is recommended to undergo level checks every three to six months, a schedule that our patient followed, supervised by his primary care physician, and occurring every six months. While lithium has a narrow therapeutic range, the neurological side effects commonly associated with chronic toxicity usually occur at higher concentrations than those observed in our patients. Nevertheless, it is crucial to recognize that serum concentrations do not always reflect the degree of toxicity [3]. It's important to also account for factors like the duration of lithium exposure, concurrent medical conditions and medications, as well as risk factors for toxicity, including suboptimal living conditions, individuals with mental health disorders, and the elderly. Elderly individuals are notably more susceptible to the effects of lower lithium doses compared to younger adults, primarily because of age-related reductions in glomerular filtration rate and a diminished volume of distribution [4]. According to the nephrology team overseeing this patient's care, hemodialysis was deemed unnecessary. Instead, the approach involved administering intravenous fluids to enhance the elimination of lithium through the kidneys while concurrently addressing the acute kidney injury. Hemodialysis is advised in cases of impaired kidney function, a lithium serum level exceeding 4 mEq/L, or if any of the following conditions are present: decreased level of consciousness, seizures, or life-threatening cardiac dysrhythmias, regardless of the specific lithium levels [5]. The cause of this patient's acute kidney injury, which triggered lithium toxicity and prompted his visit to the emergency department, remains uncertain and is most likely multifactorial. Considering that lithium has the potential to induce kidney damage, this remains a plausible explanation, though it appears less likely in light of the patient's stable use of the same dose for over three years. Moreover, it's important to note that lithium-induced nephropathy typically develops gradually and progresses to end-stage renal disease over time, which wouldn't fully account for his onset of acute kidney injury [6]. Another potential cause involves the advancement of his chronic kidney disease, initially stemming from hypertension as the root cause. However, this seems less probable given that the patient's hypertension has been effectively managed with medical treatment. Prior to the patient's presentation, it was determined that he had experienced a decline in heart function, as evidenced by a decrease of 15-20% in his ejection fraction compared to the prior measurement. Considering the known association between heart failure and chronic kidney disease (CKD), it's plausible that the development of his symptoms could be linked to the expected progression of his medical condition. Nonetheless, this perspective does not account for the family's observations, as they reported a noticeable and rapid deterioration in his condition following the introduction of spironolactone to his medication regimen. Upon examining the patient's kidney function both before and after the introduction of spironolactone, it does appear that there is a connection between the decline in renal function and the onset of symptoms as reported by the family.

Spironolactone, an antagonist of aldosterone receptors, acts on the renin-angiotensin-aldosterone system (RAAS) to enhance the excretion of sodium and water, all the while maintaining potassium levels. Spironolactone has been shown to reduce morbidity and mortality in patients with severe congestive heart failure and guidelines have recommended adding spironolactone to treatment with angiotensin-converting enzyme (ACE) inhibitors and beta blockers [7]. When prescribing for patients with concurrent heart failure and kidney disease, it's imperative to give careful consideration to specific dosing requirements. Practice guidelines recommend for patients with estimated estimated glomerular filtration rate (eGFR) of 30 to 50 mL/minute/1.73 m^2^ to begin with an initial dose of 12.5 mg once daily or every other day [8]. Doses can be doubled every four weeks if serum potassium remains less than 5 mEq/L and kidney function is stable, up to a maximum target dose of 25 mg/day. Our patient started on 25 mg daily. In a recently published case-control study, the new use of spironolactone was shown to be associated with acute renal failure [9]. Diminished renal function has been demonstrated as a potential side effect of spironolactone, despite many clinicians being unaware of this connection [10]. An increase in serum creatinine is well established as a sequelae of spironolactone; however, it is unclear as to whether this represents intrinsic kidney injury or a change in hemodynamics [11]. As indicated by the information in Table 2, there was a noteworthy decrease in our patient's renal function, as evaluated through blood urea nitrogen (BUN), creatinine, and eGFR (calculated using the 2021 CKD-EPI (Chronic Kidney Disease Epidemiology Collaboration) equation), subsequent to the administration of spironolactone, which occurred 55 days before his visit to the emergency department. His cardiologist discontinued spironolactone eight days before his presentation, and there was a slight improvement in his renal function, as evidenced by laboratory results obtained eleven days before his presentation and on the day of his presentation. The initial three columns depict his stable baseline renal function, which remained consistent over a span of three years. Fifty-seven days before his presentation, his BUN levels increased while his creatinine and eGFR values remained at their baseline levels. This suggests a probable cause other than renal damage, such as dehydration. The exact causation of this patient's renal decline remains uncertain, but it appears that spironolactone may have contributed to the situation in some way. Further research endeavors should prioritize the exploration and clarification of the mechanisms responsible for kidney injury caused by spironolactone to enhance our comprehension of this issue.

**Table 2:** Renal function prior to patient presentation to the emergency department * Number of days prior to presentation to the emergency department

No. of days*	439	183	75	57	32	11	0	Reference Range
BUN (mg/dL)	16	18	19	24	28	35	35	8.0-23
Creatinine (mg/dL)	1.59	1.55	1.55	1.53	1.79	2.29	2.11	0.67-1.17
eGFR (mL/min/1.73 sq meter)	44	45	45	45	38	28	31	≥60

Another consideration is to assess the potential influence of introducing Entresto on the patient's current presentation, which took place 88 days prior to the current visit. Entresto, introduced in 2015, is an innovative medication that merges sacubitril, a prodrug metabolized into the active LBQ657 metabolite, known for inhibiting neprilysin, with valsartan, an angiotensin II receptor blocker. Its mechanism involves the dual action of inhibiting neprilysin and blocking the angiotensin II type-1 (AT1) receptor thereby lowering blood pressure and improving heart failure. When examining Table 2, it is evident that the patient's BUN/CR remained consistent in the subsequent two blood draws, indicating good tolerance to the introduction of Entresto. This is not to dismiss the possibility that Entresto contributed to the patient's condition; however, the connection is not as apparent when examining the patient's kidney function. Since its introduction there have yet to be any case reports linking Entresto to exacerbation of chronic kidney disease; however, more research needs to be conducted to determine if such a connection exists.

## Conclusions

This case highlights the importance of maintaining an open differential diagnosis, as lithium toxicity can manifest with symptoms resembling other neurological conditions like Parkinson's disease dementia. Lithium toxicity should be taken into account in the evaluation of any patient undergoing lithium therapy who presents with neurological issues, a well-documented side effect. Additionally, it is crucial to give thorough thought to and adhere to specific dosing guidelines when prescribing new medications to patients already receiving lithium therapy and concurrently suffering from chronic kidney disease.

While spironolactone has been demonstrated to offer advantages in terms of reducing morbidity and mortality in individuals with congestive heart failure, this paper raises concerns regarding the potential for triggering exacerbation of chronic kidney disease. It is crucial for healthcare providers to be cognizant of this potential risk and to factor it into their decision-making when contemplating the prescription of spironolactone for patients with congestive heart failure who also have concomitant chronic kidney disease.
